# Little devil takes your breath away

**DOI:** 10.1007/s12471-019-1283-9

**Published:** 2019-05-10

**Authors:** M. A. C. Koole, J. A. Winkelman, A. Kaya, M. A. Beijk

**Affiliations:** 1 Department of Cardiology, Red Cross Hospital, Beverwijk, The Netherlands; 2 Department of Cardiothoracic Surgery, Amsterdam UMC, University of Amsterdam, Amsterdam, The Netherlands; 3 Department of Cardiology, Amsterdam UMC, University of Amsterdam, Amsterdam, The Netherlands

A 67-year-old female patient was referred with progressive dyspnoea on exertion. Coronary angiography was performed (Fig. 1a, b).

What is your diagnosis?

**Fig. 1:**
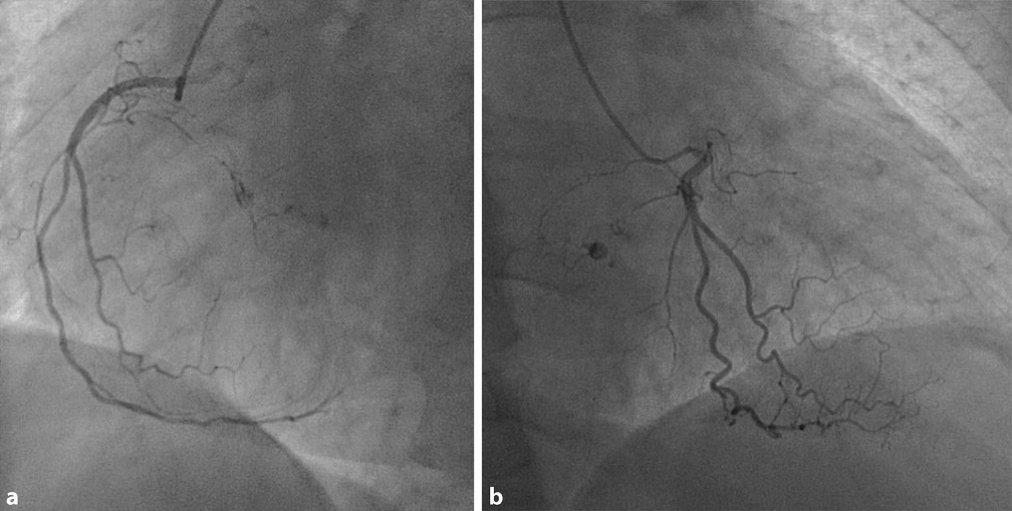
Coronary angiography of the right coronary artery. **a** Left anterior oblique view. **b** Right anterior oblique view

## Answer

You will find the answer elsewhere in this issue.

## Caption Electronic Supplementary Material

Left anterior oblique view of the right coronary artery

Right anterior oblique view of the right coronary artery

